# MRSA in Conventional and Alternative Retail Pork Products

**DOI:** 10.1371/journal.pone.0030092

**Published:** 2012-01-19

**Authors:** Ashley M. O'Brien, Blake M. Hanson, Sarah A. Farina, James Y. Wu, Jacob E. Simmering, Shylo E. Wardyn, Brett M. Forshey, Marie E. Kulick, David B. Wallinga, Tara C. Smith

**Affiliations:** 1 Department of Epidemiology, College of Public Health, University of Iowa, Iowa City, Iowa, United States of America; 2 Center for Emerging Infectious Diseases, Coralville, Iowa, United States of America; 3 Department of Biostatistics, College of Public Health, University of Iowa, Iowa City, Iowa, United States of America; 4 Institute for Agriculture and Trade Policy, Minneapolis, Minnesota, United States of America; Auburn University, United States of America

## Abstract

In order to examine the prevalence of *Staphylococcus aureus* on retail pork, three hundred ninety-five pork samples were collected from a total of 36 stores in Iowa, Minnesota, and New Jersey. *S. aureus* was isolated from 256 samples (64.8%, 95% confidence interval [CI] 59.9%–69.5%). *S. aureus* was isolated from 67.3% (202/300) of conventional pork samples and from 56.8% (54/95) of alternative pork samples (labeled “raised without antibiotics” or “raised without antibiotic growth promotants”). Two hundred and thirty samples (58.2%, 95% CI 53.2%–63.1%) were found to carry methicillin-sensitive *S. aureus* (MSSA). MSSA was isolated from 61.0% (183/300) of conventional samples and from 49.5% (47/95) of alternative samples. Twenty-six pork samples (6.6%, 95% CI 4.3%–9.5%) carried methicillin-resistant *S. aureus* (MRSA). No statistically significant differences were observed for the prevalence of *S. aureus* in general, or MSSA or MRSA specifically, when comparing pork products from conventionally raised swine and swine raised without antibiotics, a finding that contrasts with a prior study from the Netherlands examining both conventional and “biologic” meat products. In our study *spa* types associated with “livestock-associated” ST398 (t034, t011) were found in 26.9% of the MRSA isolates, while 46.2% were *spa* types t002 and t008—common human types of MRSA that also have been found in live swine. The study represents the largest sampling of raw meat products for MRSA contamination to date in the U.S. MRSA prevalence on pork products was higher than in previous U.S.-conducted studies, although similar to that in Canadian studies.

## Introduction

*Staphylococcus aureus* is estimated to cause around 185,000 cases of food poisoning annually [1]. It also can cause serious, often life threatening human infections of the bloodstream, skin, lungs, and other organs [2]. In 2003–2004, a nationally representative survey found nearly a third (28.6%) of the U.S. population was colonized in their nares with *S. aureus* [3].

This same study found the prevalence of Americans carrying *S. aureus* resistant to methicillin, or MRSA, was 1.5% [3]. While once mainly a hospital problem, MRSA infections occurring in community settings have increased in incidence over the last decade [4], with community-associated MRSA strains now constituting the most frequent cause of skin and soft tissue infections presenting to emergency departments [5], [6]. In the U.S., these community-associated MRSA (CA-MRSA) strains have been of apparent human origin. In parts of Europe, however, livestock-associated MRSA–in particular, the swine-associated ST398 strain–appears to be an important, emerging strain of human MRSA in the community [7], [8], and has been reported as a cause of human disease in several countries [9], [10], and of deaths in Spain and France [11], [12].

The finding of MRSA ST398 in farm environments has been accompanied by a rising concern regarding the presence of ST398 in the food supply, which may allow food to serve as a vehicle to transmit animal-associated MRSA into the human population. In North America, MRSA ST398 has been detected in pigs and farmers in Canada and the United States (U.S.) [13], [14], and human ST398 infections have been demonstrated in Canada [15]. As not all patients diagnosed with ST398 infections had known contact with livestock, the possibility of acquisition of ST398 via handling of contaminated pork products was suggested.

There has been less study overall of MRSA in retail meat products than in the farming environment. Studies conducted outside the U.S. have detected MRSA strains in 1.2 percent to 35.3 percent of samples from a variety of such products including retail meats in Canada [16], [17], Spain [18] and the Netherlands [19], [20]. Thirty-two percent of the MRSA strains in pork samples from one Canadian study were identified as ST398 [16], but none in the second study [17], while 50 percent from the Spanish study (1 of 2) and 85 percent of MRSA strains in the Dutch meat samples were ST398 [18], [19].

Preliminary testing of limited numbers of retail meat products in the U.S. have produced mixed results. MRSA was detected in five of 90 (5.6 percent) pork samples and one of 30 (3.3 percent) beef samples in Louisiana [21]; none of 36 meat samples (12 beef, 12 chicken, 12 pork) collected from stores in Rhode Island [22]; and two of 55 pork samples (3.6 percent) tested in an study of Iowa retail meats [23]. Only one of the MRSA strains identified in these prior studies was ST398. A recent study by Waters et al found *S. aureus* in 47 percent of 136 meat samples collected from 4 states and the District of Columbia. While ST398 strains were found in methicillin-susceptible *S. aureus* from this study, no MRSA ST398 was detected [24].

As MRSA has been found in a higher percentage of retail meats in other countries and there are significant limitations in the U.S. studies conducted to date (including variability in the methods used between studies, limiting the comparisons between states, see Table 1), we designed our study to include a higher number of samples (n = 395) of one type of meat (pork) from three geographic areas: two of them being Midwestern states among the nation's top three pork producers, and one in the outskirts of New York City, a major U.S. population center. We tested a wide range of raw pork products, both pre-packaged and individually wrapped at point of purchase from the meat counter. Products were collected from retail chain stores, some of which could be found in all three states, but most of which were part of local or regional chains. Furthermore, as no U.S. study had specifically tested potential differences in *S. aureus* contamination between conventional and “alternative” pork products (labeled and/or marketed as raised without antibiotics or raised without antibiotic growth promotants), we also included these meats in our sample.

**Table 1:** Previous research examining prevalence and molecular types of MRSA on raw meat samples.

Study and location	Meat type	Number of samples	MRSA prevalence	ST 398-associated types
[21], Louisiana	Beef	30	3.3%	0%
	Pork	90	5.6%	0%
[22], Rhode Island	Beef	12	0	N/A
	Pork	12	0	N/A
	Chicken	12	0	N/A
[23], Iowa	Pork	55	3.6%	50%
	Beef	29	0	N/A
	Chicken	45	0	N/A
	Turkey	36	0	N/A
[This study], Iowa, Minnesota, New Jersey	Pork	395	6.6%	26.9%
[24], Arizona, California, Florida, Illinois, Washington, DC	Pork	26	3.8%	0
	Beef	38	2.6%	0
	Chicken	46	0	N/A
	Turkey	26	3.8%	0
[16], Canada	Pork	402	7.7%	32%
[17], Canada	Pork	230	9.6%	0
	Beef	198	5.6%	0
	Chicken	250	1.2%	0
[20], Netherlands	Pork	64	3.1%	50%
	Beef	15	0	N/A
[19] *, Netherlands	Pork	309	10.7%	97%
	Beef	395	10.6%	60%
	Chicken	520	16.0%	89%
	Turkey	116	35.5%	93%
[18] #, Spain	Pork	55	1.8%	100%
	Chicken	148	0.7%	0
	Turkey	10	0	N/A

*Samples of veal, lamb/mutton, fowl, and game were also collected in this study but not included in the table.

# Samples of rabbit, minced meat, veal, lamb, and wild game were also collected in this study but not included in the table.

## Materials and Methods

### Sample collection and culture

Three hundred ninety-five raw pork samples were collected from 36 retail food stores in Iowa, Minnesota, and New Jersey in four rounds of sampling carried out at weekly intervals in September and October 2010. Stores were chosen to include a mix of national and regional supermarkets and retail chains, specialty food markets and cooperative grocery stores. Pork cuts collected included pork chop, ground pork, riblets, ribs, sausage, blade steak, cube steaks, pork loin, pork roast, and pork cutlet; all were either identified as fresh on the package label and did not appear to have been previously frozen or were verbally confirmed as fresh at the meat counter. Pork packages were double-bagged upon purchase to avoid cross-contamination, shipped overnight on blue ice following Federal Express guidelines for shipping perishable foods, and analyzed within 24 hours of purchase.

Upon arrival at the laboratory, packaging was removed using a sterile razor blade and pork samples were transferred into stomacher bags (VWR, West Chester, PA) with sterile tongs. Sample weight was recorded and 100 g was used as a minimal sample size. Two hundred and fifty mL of sterile 0.1% peptone broth was added to the sample by sterile graduated cylinder. Samples were then mixed vigorously by hand for one minute. A 30 ml aliquot from the peptone wash was added to 30 ml of Baird Parker broth (2× concentration) with tellurite enrichment (Sigma products – Sigma-Aldrich, St. Louis MO) in a 250 ml sterile Erlenmeyer flask and incubated at 35°C for 18–24 hours. After enrichment, 10 µl was plated onto Baird Parker agar (BPA) with EY tellurite enrichment (BD) and incubated at 35°C 18–24 hours for growth. Ten µL were also plated onto CHROMagar MRSA plates (BD) and incubated at 35°C 24–48 hours and examined for growth. Presumptive *S. aureus* (black colonies with clear halos on BPA) and presumptive MRSA (mauve colonies on CHROMagar) were confirmed by examining their appearance on Gram stain, and by doing the catalase test, the tube coagulase test and the *S. aureus* latex agglutination assay (Pastorex Staph-plus, Bio-Rad). Methicillin resistance was assessed by testing for the presence of penicillin binding protein (PBP2') (MRSA latex agglutination test, Oxoid Ltd., Hants, UK) and confirmed with *mecA* polymerase chain reaction (PCR). *S. aureus* isolates were stored at −80°C.

### Molecular testing

One MRSA isolate per positive specimen was subjected to molecular typing. Genomic DNA was extracted using the Wizard Genomic DNA preparation kit (Promega). The presence of *mecA* and PVL were determined by PCR [25], [26]. Multi locus sequence typing (MLST) was performed on a subset of isolates [27]. *spa* typing was carried out using the alternate forward primer described in [28] and the reverse primer noted on the Ridom website (http://www.ridom.de/doc/Ridom_spa_sequencing.pdf). All molecular procedures employed known positive and negative controls.

### Antimicrobial susceptibility testing (AST)

All presumptive MRSA isolates were also tested for antimicrobial susceptibility by the broth dilution method described by the Clinical and Laboratory Standards Institute [29]. Isolates were tested for susceptibility to the following 10 antibiotics using this method: oxacillin, tetracycline, erythromycin, clindamycin, trimethoprim-sulfamethoxazole, quinupristin/dalfopristin, levofloxacin, linezolid, daptomycin, and vancomycin. Mupirocin resistance and inducible clindamycin resistance were also examined [30].

### Survey/data analysis

Analyses were conducted for two groups: all *S. aureus* (MSSA and MRSA together) and MRSA specifically. Exact 95% confidence intervals (CI) were calculated for unadjusted prevalence rates. Heterogeneity of *S. aureus* or MRSA positivity among categorical variables was addressed using Pearson's chi-square test with Yates' correction or by Fisher's exact test. To control for correlation of samples within each store, cluster-adjusted odds ratios (OR) and CIs were calculated using PROC GENMOD in SAS 9.2 (SAS Institute, Cary, NC), with an exchangeable correlation matrix structure. Significance was assessed at alpha = 0.05.

## Results

### Prevalence of *S. aureus* in pork samples

Three hundred ninety-five pork samples were collected from a total of 36 stores in Iowa, Minnesota, and New Jersey. *S. aureus* was isolated from 256 samples (64.8%, 95% CI 59.9%–69.5%). *S. aureus* prevalence in retail pork from Iowa, Minnesota, and New Jersey was 59.7% (74/124, 95% CI 50.5%–68.4%), 72.3% (102/141, 95% CI 64.2%–79.5%), and 61.5% (80/130, 95% CI 52.6%–69.9%), respectively. These differences among states were not statistically significant (χ^2^ = 5.548, degrees of freedom (df) = 2, p = 0.062; adjusted for clustering within stores, p = 0.306). Likewise, no significant difference was observed for *S. aureus* prevalence between conventional and alternative pork samples (χ^2^ = 3.482, df = 1, p = 0.062; adjusted OR 1.56, 95% CI 0.86—2.83). Of 300 conventional pork samples, *S. aureus* was isolated from 202 samples (67.3%, 95% CI 61.7%–72.6%) while *S. aureus* was isolated from 54/95 (56.8%, 95% CI 46.3%–67.0%) of pork labeled raised without antibiotics or raised without antibiotic growth promotants.

### Prevalence of MRSA in pork samples

Twenty-six MRSA positive samples were identified (6.6%, 95% CI 4.3%–9.5%). Prevalence was 8.1% in Iowa (10/124, 95% CI 3.9%–14.3%); 7.1% (10/141, 95% CI 3.5%—12.7%) in Minnesota; and 4.6% (6/130, 95% CI 1.7%–9.8%) in New Jersey (χ^2^ = 1.321, df = 2, p = 0.517; adjusted for clustering within stores, p = 0.409). MRSA-positive samples were collected from 41.7% (15/36) of the stores included in the study. Similar prevalences of MRSA-positivity were found in conventional pork (6.3% [19/300], 95% CI 3.9%—9.7%) and in alternative pork (7.4% [7/95], 95% CI 3.0%–14.6%; adjusted OR 0.93, 95% CI 0.276—3.16). Six of the seven MRSA positive alternative pork samples came from the same retail chain (17.1% [6/35] of samples from the chain) in two different states and four from the same store (21.1% [4/19] of samples from the store), the largest number of MRSA isolates associated with any one store in this study. Of the six MRSA isolates from this chain, five (83%) were *spa* type t008.

**Figure 1:**
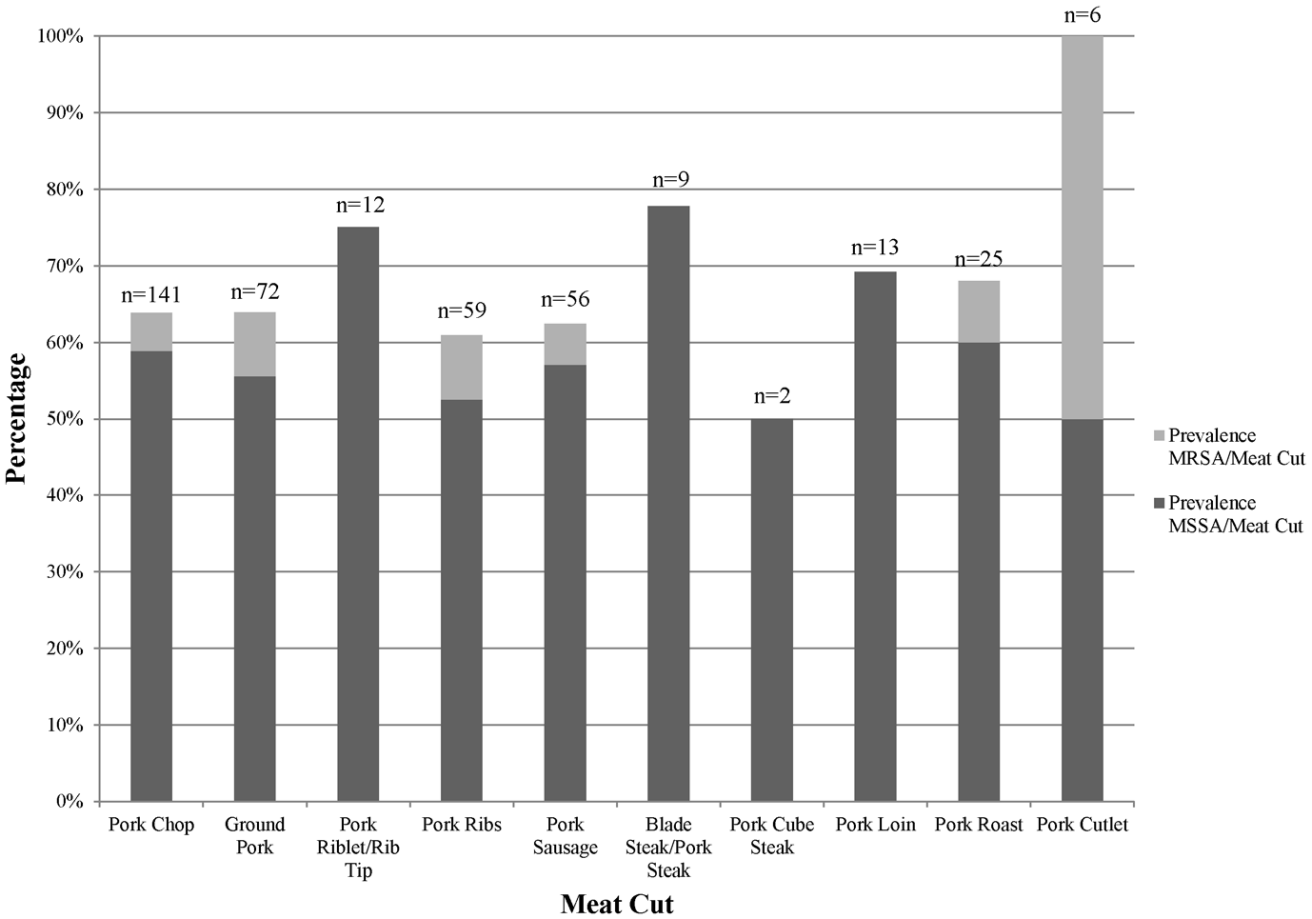
Prevalence of MRSA and MSSA in each type of meat cut. Total number of samples of each type of meat cut are noted.

By pork cut, MRSA prevalence ranged from 0% (riblets, blade steak, cube steak, pork loin) to 50% (pork cutlet). In all types of pork products, on the other hand, prevalence of MSSA equaled or exceeded 50% of samples (see Figure 1). No statistically significant differences in *S. aureus* or MRSA prevalence were seen between different cuts of pork (p = 0.725 [Pearson's Chi-square test] and p = 0.133 [Fisher's exact test] for *S. aureus* and MRSA, respectively).

### Antibiotic resistance

All 26 isolates identified as MRSA via positive PBP2' latex tests and *mecA* PCR were subjected to AST. Twenty-two (84.6%) were resistant to oxacillin; interestingly, four were found to be susceptible to oxacillin by broth microdilution, despite harboring the *mecA* gene, as we have seen in previous research [31]. Seventeen (65.4%) were resistant to tetracycline, 10 (38.5%) were resistant to erythromycin and 9 (34.6%) were resistant to clindamycin. One isolate was resistant only to TMP-SMX; another isolate was resistant to oxacillin, tetracycline, clindamycin, erythromycin, and quinupristin-dalfopristin (see Table 2). Twenty isolates (76.9%) were resistant to two or more antibiotics and ten isolates (38.5%) were resistant to three or more antibiotics tested.

**Table 2:** Description of MRSA isolates from pork samples.

Isolate	Meat type	Meat Cut	Resistance profile	PVL	*mecA*	*spa* type
**IA-6**	C	Ribs	O	−	+	t803
**IA-61**	C	Pork Cutlet	O, T, Cl, E	−	+	t078
**IA-63**	C	Pork Cutlet	O, T, Cl	−	+	t034
**IA-77**	C	Ribs	T	−	+	t8413
**IA-88**	C	Ground Pork	O	−	+	t2922
**IA-91**	C	Sausage	O, T	−	+	t034
**IA-97**	C	Pork Cutlet	O, T, Cl	−	+	t034
**IA-111**	C	Pork Chop	O, Cl, E	−	+	t002
**IA-112**	C	Ribs	O, T, Cl, E, Q/D	−	+	t002
**IA-121**	RWA	Ground Pork	O, T	−	+	t011
**MN-48**	C	Pork Roast	O, T, Cl	−	+	t034
**MN-49**	C	Pork Roast	O, T	−	+	t002
**MN-81**	C	Ground Pork	O, T	−	+	t002
**MN-82**	C	Pork Chop	T	−	+	t094
**MN-94**	C	Pork Chop	TMP-SMX	−	+	t094
**MN-113**	RWA	Ground Pork	O, T, E	−	+	t008
**MN-119**	C	Pork Chop	O, T	−	+	t002
**MN-135**	C	Sausage	O, T, Cl	−	+	t034
**MN-138**	RWA	Ground Pork	O, E	+	+	t008
**MN-144**	C	Ribs	O, T, Cl, E	−	+	t002
**NJ-34**	RWA	Pork Chop	O, E	+	+	t008
**NJ-68**	RWA	Pork Chop	O, E	+	+	t008
**NJ-73**	C	Sausage	O, E	+	+	t008
**NJ-92**	C	Pork Chop	T	−	+	t273
**NJ-101**	RWA	Ribs	O, T	−	+	t034
**NJ-102**	RWA	Ground Pork	O, Cl, E	+	+	t008

IA: Iowa; MN: Minnesota; NJ: New Jersey. RWA: product labeled as “raised without antibiotics”. C: conventional meats. Antibiotic resistance is denoted as follows: O: oxacillin; T: tetracycline; E: erythromycin; Cl: clindamycin; TMP-SMX: trimethoprim/sulfamethoxazole; Q/D: quinupristin-dalfopristin.

### Molecular typing

All MRSA isolates were subjected to *spa* typing. Six isolates (23.1%) were t034, and 1 (3.8%) was t011, both associated with ST398. Six isolates (23.1%) each were t002 (associated with USA100/ST5) and t008 (associated with USA300/ST8). Other types included t094 (n = 2); t078 (n = 1); t273 (n = 1); t803 (n = 1); t2922 (n = 1), and a new *spa* type, t8314 (n = 1). Two of these isolates, both from Minnesota pork samples, were *spa* type t094, but were from different cuts of pork purchased from different stores and were different lines of meat, though they were from the same brand. t8314 was a novel *spa* type and also corresponded to a new sequence type (ST2007). Five isolates (19.2%) were found to carry PVL, a genotype epidemiologically associated with increased virulence [32], [33]; all were *spa* type t008 (see Table 2). A similar percentage of both conventional and alternative pork harbored *spa* types associated with ST398 (2/7, 28.6% in alternative samples; 5/19, 26.3% in conventional samples). However, 4/5 (80%) of the PVL-positive t008 isolates were found in alternative pork samples.

## Discussion

This study confirms the presence of *S. aureus* on raw pork products in the United States, regardless of whether produced conventionally or from animals raised without antibiotics/antibiotic growth promotants. No statistically significant differences were observed when comparing *S. aureus* (whetherMSSA or MRSA) positivity on conventional and alternative pork products.

This is in contrast to findings from the Netherlands, where a lower prevalence of MRSA in meat from “biological” reared chickens (raised using no growth promotants) and wild fowl and game was found, in comparison to the prevalence of MRSA found in conventionally raised poultry in the same study [19], suggesting a link between antibiotic use and prevalence of MRSA on meat products in Europe. This was also shown in a study of U.S. pigs, where MRSA was found in four of nine “conventional” pig farms, but was absent from pigs raised without antibiotics (Smith, unpublished findings). It is possible that a difference in MRSA contamination between conventional and alternative meats does not exist in our sampling areas. Alternatively, in the U.S., it is possible that a link between on-farm antibiotic use and MRSA on meat products may be obscured by human contamination of meat with MRSA post-slaughter, as human carriage of MRSA in the U.S. is approximately 50 times higher than what has been reported in the Netherlands (1.5% vs. .03%) [34]. However, given that 85.7% (6/7) of the MRSA-positive alternative pork samples in our study came from the same retail chain in two different states and four from the same store, there could be other explanations for our findings. Both antibiotic claims used to identify alternative products for this study, “raised without antibiotics” and “raised without antibiotic growth promotants”, are not typically verified by an independent third party, unlike USDA-certified Organic meat products, which are also raised without antibiotics. While certified organic pork products were available at some of the stores included in this study, they were not available in an appropriate form for our testing, e.g., they were smoked, frozen or pre-cooked.

Additionally, similar to meats raised conventionally, pork raised without antibiotics can be contaminated with MRSA at the processing plant, either from contaminated products processed on the same equipment or by colonized workers. Processing equipment must be cleaned out between runs of certified organic and non-certified organic meats, which may help to prevent this type of cross-contamination (http://www.mda.state.mn.us/en/sitecore/content/Global/MDADocs/food/organic/organicmeatprod.aspx). Two Dutch studies showed MRSA colonization of slaughterhouse workers: one swine-based study that found MRSA ST398 and one broiler-focused study [35], [36]. In both studies transmission appeared to go from animals to humans, but exact transmission routes were not determined. To our knowledge, no similar research has been published in the U.S.

Overall, MRSA prevalence on pork products was similar to what has been found in other studies conducted in Canada, but higher than that previously identified in studies conducted in the U.S. [16], [17], [21], [22]. Strengths of our study include the sampling of three different geographic areas and the inclusion of both urban and rural areas, as well as the inclusion of a wide variety of pork cuts and brands, both conventional and raised without antibiotics. This study also represents the largest sampling of raw meat products for MRSA contamination carried out to date in the U.S.

A significant proportion of MRSA isolates (26.9%) were *spa* types associated with ST398 (t034, t011), a “livestock-associated” strain [37]. An additional 23.1% each were *spa* types t002 and t008, common human types that have also been found in live swine [13], [38]. A limitation of this study is our inability to determine the ultimate origin of these isolates, e.g., whether they are a result of contamination from the farming environment, human contamination post-slaughter, or both. Previous research has shown that there is a poultry-adapted ST5 lineage (a type which contains *spa* type t002) that originated in humans and subsequently spread amongst commercial poultry [39]; a similar cross-species transfer event could have occurred in American swine herds, resulting in a swine-adapted t002 strain. Additional genomic studies are necessary to examine this possibility.

Also unknown is the role that MRSA contamination of raw meats in the U.S., now confirmed by several studies [21], [23], [24], may play in the overall ecology and transmission of this organism. Bacterial contamination was not quantitated in this study; as such, the amount of initial colonies present on the pork samples is unknown. Also unknown is the frequency of MRSA transmission to humans, via colonization or infection from food service professional and consumer handling and consumption of raw, undercooked and cooked MRSA-positive meat. A study in the Netherlands examining MRSA carriage by food handlers did not find any MRSA-positive individuals, even though some had been handling meat products which were contaminated with MRSA [40]. However, it is difficult to directly apply these findings to consumers, who may not receive as much training in proper food handling. Future studies are necessary to examine this aspect.
